# Noradrenergic activation of the basolateral amygdala modulates the consolidation of object-in-context recognition memory

**DOI:** 10.3389/fnbeh.2014.00160

**Published:** 2014-05-08

**Authors:** Areg Barsegyan, James L. McGaugh, Benno Roozendaal

**Affiliations:** ^1^Department of Cognitive Neuroscience, Radboud University Medical CentreNijmegen, Netherlands; ^2^Donders Institute for Brain, Cognition and Behaviour, Radboud UniversityNijmegen, Netherlands; ^3^Department of Neuroscience, Section Anatomy, University Medical Center GroningenGroningen, Netherlands; ^4^Center for the Neurobiology of Learning and Memory, Department of Neurobiology and Behavior, University of CaliforniaIrvine, CA, USA

**Keywords:** norepinephrine, emotional arousal, episodic-like memory, object recognition, hippocampus

## Abstract

Noradrenergic activation of the basolateral complex of the amygdala (BLA) is well known to enhance the consolidation of long-term memory of highly emotionally arousing training experiences. The present study investigated whether such noradrenergic activation of the BLA also influences the consolidation of object-in-context recognition memory, a low-arousing training task assessing episodic-like memory. Male Sprague–Dawley rats were exposed to two identical objects in one context for either 3 or 10 min, immediately followed by exposure to two other identical objects in a distinctly different context. Immediately after the training they received bilateral intra-BLA infusions of norepinephrine (0.3, 1.0, or 3.0 μ g) or the β-adrenoceptor antagonist propranolol (0.1, 0.3, or 1.0 μ g). On the 24-h retention test, rats were placed back into one of the training contexts with one copy of each of the two training objects. Thus, although both objects were familiar, one of the objects had not previously been encountered in this particular test context. Hence, if the animal generated a long-term memory for the association between an object and its context, it would spend significantly more time exploring the object that was not previously experienced in this context. Saline-infused control rats exhibited poor 24-h retention when given 3 min of training and good retention when given 10 min of training. Norepinephrine administered after 3 min of object-in-context training induced a dose-dependent memory enhancement, whereas propranolol administered after 10 min of training produced memory impairment. These findings provide evidence that post-training noradrenergic activation of the BLA also enhances the consolidation of memory of object-in-context recognition training, enabling accuracy of episodic-like memories.

## Introduction

It has long been known that noradrenergic activation of the basolateral complex of the amygdala (BLA) is crucially involved in strengthening the consolidation of long-term memory (McGaugh, 2000, 2004; McGaugh and Roozendaal, 2002). Norepinephrine or a β-adrenoceptor agonist infused into the BLA immediately post-training enhances the retention of many different types of emotionally arousing training experiences, including inhibitory avoidance (Introini-Collison et al., 1991; Ferry et al., 1999), contextual fear conditioning (LaLumiere et al., 2003; Huff et al., 2005), water-maze spatial training (Hatfield and McGaugh, 1999), and extinction of contextual fear conditioning (Berlau and McGaugh, 2006). In contrast, intra-BLA infusions of a β-adrenoceptor antagonist impair the consolidation of memory for these training experiences (Hatfield and McGaugh, 1999; Miranda et al., 2003). Extensive evidence indicates that such nor-adrenergic manipulation of amygdala activity, in turn, facilitates information storage processes in other brain regions known to be involved in memory processing, including the hippocampus, caudate nucleus, and insular cortex (McGaugh, 2004; Roozendaal and McGaugh, 2011).

Prior studies investigating the involvement of BLA noradrenergic activity in regulating memory consolidation have, however, primarily employed highly arousing training conditions that are known to induce the release of high levels of norepinephrine within the amygdala (Quirarte et al., 1998; Hatfield et al., 1999; McIntyre et al., 2002). The aversive nature of highly arousing conditions inherent to, for example, water maze or inhibitory avoidance training invariably prompts survival-driven behaviors in animals when swimming for safety or escaping footshocks. While these behavioral paradigms are highly effective for inducing learning and result in adaptive strategies for an organism, they do not necessarily address forms of learning and memory involving low-arousing training conditions that are not fearful or aversive in nature. In contrast, most human studies investigating the role of the noradrenergic system in memory modulation have used experimental paradigms incorporating emotionally arousing and neutral pictures, stories, words, or movie clips (Cahill et al., 1994; Southwick et al., 2002; Kensinger and Corkin, 2003; Van Stegeren et al., 2005), stimuli that do not readily evoke feelings of imminent danger or fear in participants. Consequently, it is difficult to directly compare findings from animal and human studies without adequately addressing this existing disparity. Furthermore, it is also difficult to ascertain whether memory enhancement resulting from high levels of emotional arousal enables memories with greater degree of accuracy or whether such memories are simply stronger in their emotionality. In animal studies utilizing footshock, for example, increased freezing may, without proper controls, be interpreted as stronger memory of the details of an experience or imply a greater degree of discrimination by the animal of a dangerous environment.

With these considerations in mind, in a previous study we trained rats for novel object recognition, a task known to induce only low levels of emotional arousal (Roozendaal et al., 2006; Maroun and Akirav, 2008), and found that norepinephrine administration into the BLA dose-dependently enhanced 24-h memory, while the β-adrenoceptor antagonist propranolol impaired memory of this training (Roozendaal et al., 2008). These findings thus indicate that noradrenergic activation of the BLA is also able to modulate the consolidation of non-aversive or non-fearful memories and provide evidence that this neuromodulatory system ensures lasting memories of significant experiences with varying degrees of emotionality. Standard object recognition training and memory for an object itself has been shown to depend primarily on cortical areas (Ennaceur and Aggleton, 1997; Bermudez-Rattoni et al., 2005; Albasser et al., 2009; Roozendaal et al., 2010; Banks et al., 2014; Bermudez-Rattoni, 2014). However, recognition memory as an integrated whole is more complex and encompasses a number of additional components, such as an item's associations with its context, place, etc., and involves a network of interacting brain regions (Bussey et al., 1999, 2000; Wan et al., 1999). The present study investigated whether the BLA noradrenergic system also plays a modulatory role in this more complex form of recognition memory by examining the effects of post-training intra-BLA infusions of norepinephrine or propranolol on memory of hippocampus-dependent object-in-context recognition training. This modified version of the standard object recognition task has been developed to assess episodic-like memory in rats where two similar presentation events are distinguished by the contexts in which they appear (Dix and Aggleton, 1999; Eacott and Norman, 2004; Balderas et al., 2008). Specifically, during retention testing, rats are presented with two familiar objects, only one of which had not been encountered previously in the current context, although the context itself was familiar. Thus, the configuration of object and context is novel. If noradrenergic activation of the BLA facilitates the integration of the object and context components into long-term memory, rats would be expected to explore preferentially the object that appears in a novel context over an object that is in a familiar context.

## Materials and methods

### Subjects

Male Sprague–Dawley rats (280–320 g at the time of surgery) from Charles River Breeding Laboratories (Kisslegg, Germany) were housed individually in a temperature-controlled (22°C) vivarium room and maintained on a 12-h/12-h light/dark cycle (07:00–19:00 h lights on) with *ad libitum* access to food and water. Training and testing were performed during the light phase of the cycle, between 10:00 and 15:00 h. All experimental procedures were in compliance with the European Communities Council Directive on the use of laboratory animals of November 24, 1986 (86/609/EEC) and approved by the Institutional Animal Care and Use Committee of the University of Groningen, the Netherlands.

### Cannula implantation

After an acclimatization period of at least 1 week, the animals received surgical implantation of cannulae aimed at the BLA according to a standardized protocol (Fornari et al., 2012). They were anesthetized (subcutaneous injection) with a mixture of ketamine (37.5 mg/kg body weight; Alfasan) and dexmedetomidine (0.25 mg/kg; Orion) and received the non-steroidal analgesic carprofen (4 mg/kg; Pfizer). The skull was positioned in a stereotaxic frame (Kopf Instruments, Tujunga, CA), and two stainless-steel guide cannulae (15 mm; 23 gauge; Component Supply Co/SKU Solutions, Fort Meade, FL) were implanted bilaterally with the cannula tips 2.0 mm above the BLA. The coordinates were based on the atlas of Paxinos and Watson (2005): anteroposterior, −2.8 mm from Bregma; mediolateral, ±5.0 mm from midline; dorsoventral, −6.5 mm from skull surface; incisor bar −3.3 mm from interaural. The cannulae were fixed to the skull with two anchoring screws and dental cement. Stylets (15-mm-long 00-insect dissection pins) inserted into each cannula to maintain patency were removed only for the drug infusions. After surgery, the rats were administered atipamezole hydrochloride (2.5 mg/kg; Orion) to reverse anesthesia and subsequently injected with 3 ml of sterile saline to facilitate clearance of drugs and prevent dehydration. They were allowed to recover for 10 days before initiation of training.

### Object-in-context recognition apparatus and procedures

The animals were trained and tested in two wooden open-field boxes (40 w × 40 d × 40 h cm) placed next to one another in a dimly illuminated experimental room (60 lux). One box had gray inner walls and the floor was covered with standard sawdust. The other box had four black-and-white striped walls and a floor covered with corncob bedding material. The combination of black-and-white striped walls and different bedding material made this a distinctly different contextual environment from the other box. The objects to be discriminated were white glass light bulbs (6 cm diameter, 11 cm length) and transparent glass vials (5.5 cm diameter, 5 cm height) secured to the floor of the boxes with Velcro tape.

#### Behavioral task

**Figure 1:**
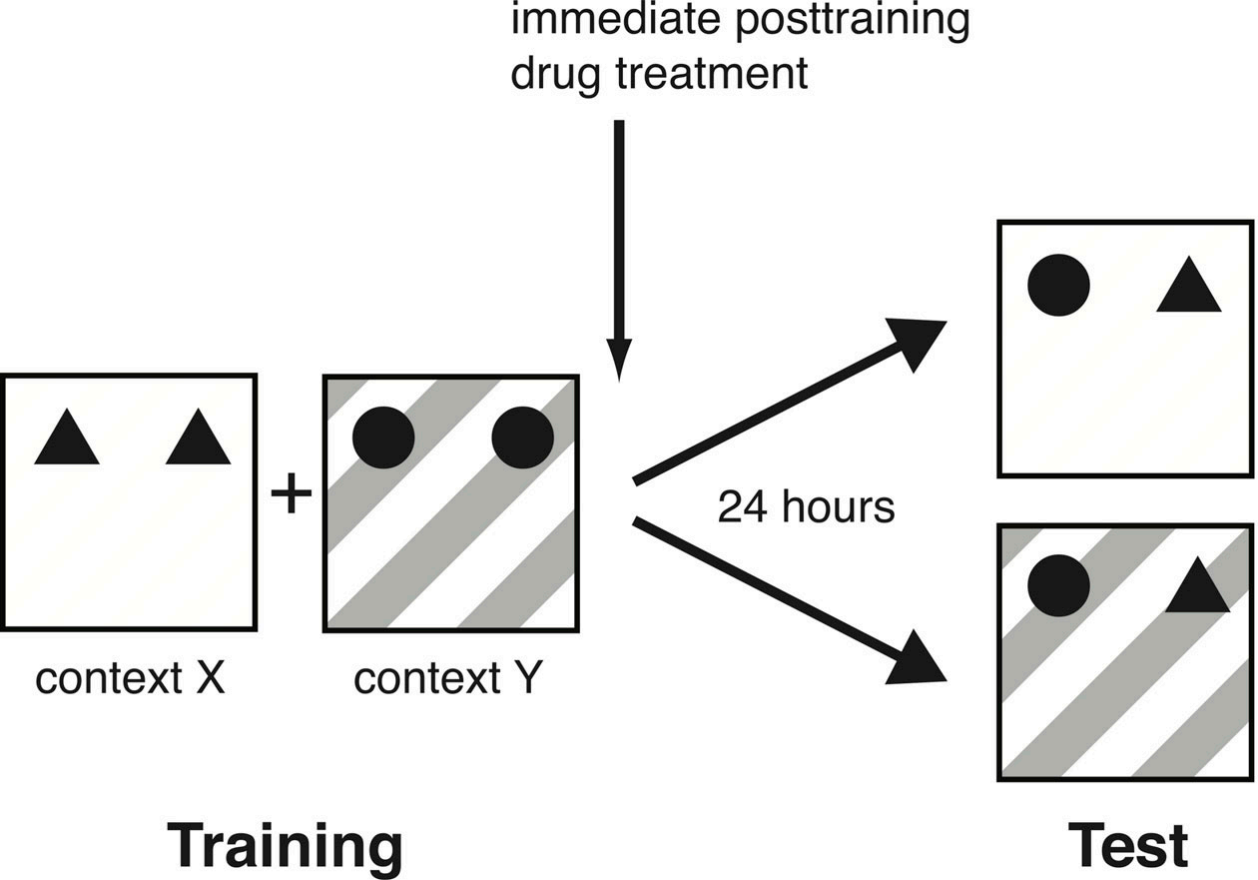
**Schematic diagram of the experimental design**. On the training session, rats were placed in one distinctive context (X) with two identical objects (A1 and A2), followed by another distinctive context (Y) with two other identical objects (B1 and B2). They were trained in each context for 3 min (norepinephrine experiment) or 10 min (propranolol experiment). On the 24-h retention test, they were placed in context X or Y with one copy of both training objects (A3 and B3) and were allowed to explore them for 3 min.

All rats were handled 1–2 min each day for 5 days prior to training. On the training session, the rat was placed in one box (context X), facing the wall, at the opposite end from the objects. The animal was allowed to explore two identical objects (A1 and A2) for either 3 or 10 min. The 3-min training session was used for the norepinephrine-treatment groups in order to assess memory enhancement and 10 min of training for the propranolol-treatment groups in order to assess memory impairment (Okuda et al., 2004; Bermudez-Rattoni et al., 2005; Roozendaal et al., 2006). Immediately after the first context exposure, the animal was placed in the adjacent box (context Y) containing two other identical objects (B1 and B2) for 3 or 10 min. To avoid the presence of olfactory cues, the bedding material was stirred and the objects were thoroughly cleaned with 70% ethanol after each rat. On the 24-h retention test, the rat was placed in either context X or Y with one copy of both training objects (A3 and B3) and was allowed to explore them for 3 min (Figure 1). Thus, although both objects were familiar, one of the objects had not been seen in this particular test context. Hence, if the animal remembered the association between an object and its context on the retention test, it would spend significantly more time exploring the object that was in a novel environment. During the training phase, context X and Y, as well as context-object combinations were counterbalanced. Similarly, during the test phase, context-object combinations were counterbalanced and randomized. Rats' behavior during training and test was recorded with a video camera mounted above the experimental apparatus. Videos were analyzed off-line by a trained observer who was unaware of treatment condition. The time spent exploring each object and the total time spent exploring both objects were recorded for both the training and test session. Exploration of an object was defined as pointing the nose to the object at a distance of <1 cm and/or touching it with the nose (Okuda et al., 2004). Turning around, climbing, or sitting on an object was not considered exploration. The discrimination index used to assess memory was calculated as the difference in time exploring the novel and familiar object, expressed as the ratio of the total time spent exploring both objects [i.e., (Time Novel − Time Familiar/Time Novel + Time Familiar) × 100%]. Rats (*N* = 6) showing a total exploration time of less than 10 s on training were removed from further analyses because previous data indicate that such rats do not adequately acquire the task (Okuda et al., 2004).

### Drug administration

Norepinephrine (0.3, 1.0, or 3.0 μ g; Sigma-Aldrich) or the β-adrenoceptor antagonist propranolol (0.1, 0.3, or 1.0 μ g; Sigma-Aldrich) was dissolved in saline and administered into the BLA immediately after the training phase. Bilateral infusions of drug or an equivalent volume of saline were given via 30-gauge injection needles connected to 10-μ l Hamilton microsyringes by polyethylene (PE-20) tubing. The injection needles protruded 2.0 mm beyond the cannula tips and a 0.2-μ l injection volume per hemisphere was infused over a period of 30 s by an automated syringe pump (Stoelting). The injection needles were retained within the cannulae for an additional 20 s to maximize diffusion and to prevent backflow of drug into the cannulae. The infusion volume was based on previous findings from our laboratory indicating that similar infusions into the adjacent central amygdala do not affect memory consolidation (Roozendaal and McGaugh, 1996, 1997). All drug solutions were freshly prepared before each experiment.

### Cannula placement verification

**Figure 2:**
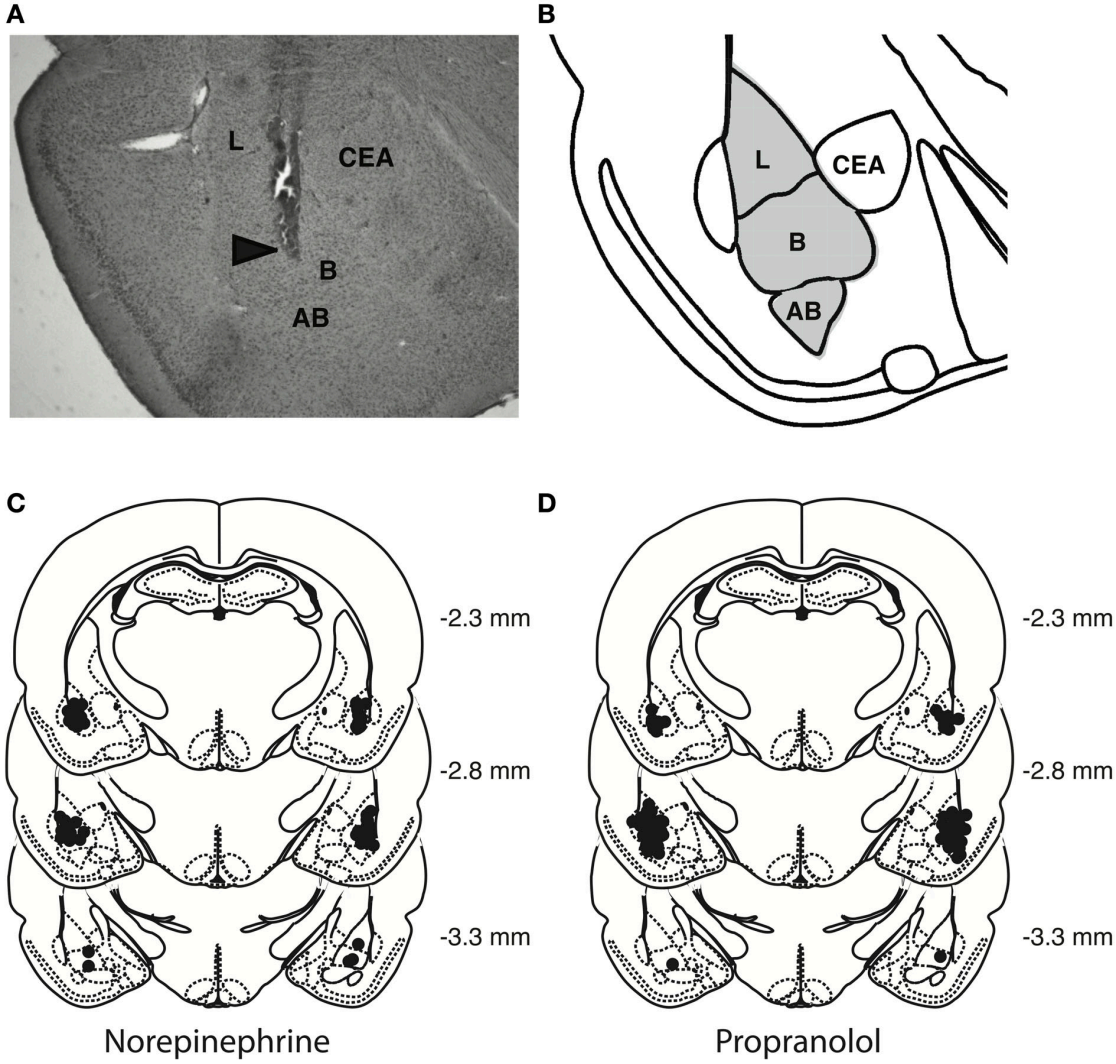
**Histological analyses. (A)** Representative photomicrograph illustrating placement of a cannula and needle tip in the BLA. Arrow points to needle tip. **(B)** The gray area in the diagram represents the different nuclei of the BLA: the lateral nucleus (L), basal nucleus (B), and accessory basal nucleus (AB). CEA, central nucleus of the amygdala. **(C,D)** Location of infusion needle tips of all rats included in the final analyses.

Rats were deeply anesthetized with an overdose of sodium pentobarbital and perfused transcardially with 0.9% saline followed by 4% formaldehyde. The brains were removed and stored in 4% formaldehyde. At least 24 h before sectioning, brains were placed in a 20% sucrose solution in water for cryoprotection. Coronal sections of 50 μm were cut on a cryostat, mounted on gelatin-coated slides, stained with cresyl violet and examined by light microscopy by an observer blind to drug treatment. Data of 21 rats with injection needle placements outside the BLA or with extensive tissue damage at the injection needle site were excluded from analyses. Figure 2 shows a representative photomicrograph of a needle track terminating within the BLA and depicts the location of infusion needle tips of all rats included in the final analyses.

### Statistics

Data are expressed as the mean ± SEM. Total object exploration during the training phase was analyzed with Two-Way analysis of variance (ANOVA) with training context (2 levels) as within-subject variable and later drug treatment groups (4 levels) as between-subject variable. Unpaired *t*-tests were used to determine whether rats had a preference for either of the training objects. The discrimination index and total object exploration time during the retention test trial were analyzed with One-Way ANOVA for drug treatment (4 levels), followed by *post-hoc* comparison tests, when appropriate. One-sample *t*-tests were used to determine whether the discrimination index was different from zero and thus whether learning had occurred. A probability level of <0.05 was accepted as statistical significance.

## Results

These experiments investigated whether an activation or inhibition of noradrenergic transmission within the BLA modulates the consolidation of object-in-context recognition memory. Independent groups of rats were trained on the object-in-context recognition task and received post-training intra-BLA infusions of either norepinephrine or propranolol. Retention was tested 24 h later. If the norepinephrine administration enhances object-in-context recognition memory in rats receiving 3 min of training, on the retention test, they will spend significantly more time exploring the object that is presented in a novel context. Similarly, if a blockade of endogenous noradrenergic activity with propranolol in animals receiving 10 min of training impairs memory consolidation, they will spend less time exploring the object presented in the novel context.

### Effect of norepinephrine in the basolateral amygdala on memory consolidation of object-in-context training

#### Training session

A Two-Way repeated-measures ANOVA for total object exploration time during the two 3-min context exposures revealed a significant trial effect [*F*_(1, 48)_ = 21.37; *P* < 0.0001], but no difference between later drug treatment groups [*F*_(3, 48)_ = 1.71; *P* = 0.18] or interaction between both factors [*F*_(3, 48)_ = 0.90; *P* = 0.45]. The average time spent exploring the two objects during the first context exposure was 18.9 ± 1.1 s, and 14.0 ± 0.8 s for the second context exposure (paired *t*-test: *P* < 0.0001; *N* = 52), demonstrating that they spent significantly more time exploring the objects during their first context exposure. Rats did not show any difference in their exploration of the two different objects. Table 1 shows the object exploration time during the two context exposures for the different post-training drug treatment groups.

**Table 1:** **Total object exploration time**.

**Experiment**	**Dose**	**1st training context**	**2nd training context**	**Retention**
Norepinephrine	Saline (*N* = 13)	14.8 ± 1.9^a^	12.6 ± 1.6^a^	12.1 ± 0.9^a^
	0.3 mg (*N* = 12)	20.0 ± 2.3	15.1 ± 1.9	15.2 ± 1.1
	1.0 mg (*N* = 15)	21.7 ± 2.1	14.8 ± 1.4	14.4 ± 1.0
	3.0 mg (*N* = 12)	18.5 ± 2.3	13.3 ± 1.3	13.0 ± 0.9
Propranolol	Saline (*N* = 12)	29.7 ± 2.5^b^	23.7 ± 4.7^b^	12.6 ± 1.4^a^
	0.1 mg (*N* = 10)	30.7 ± 3.2	20.7 ± 2.8	11.7 ± 1.2
	0.3 mg (*N* = 10)	27.3 ± 3.0	18.9 ± 2.0	12.5 ± 1.7
	1.0 mg (*N* = 9)	28.9 ± 3.1	18.6 ± 4.5	11.2 ± 1.6

a Trial duration = 3 min

b Trial duration = 10 min

#### Retention test trial

**Figure 3:**
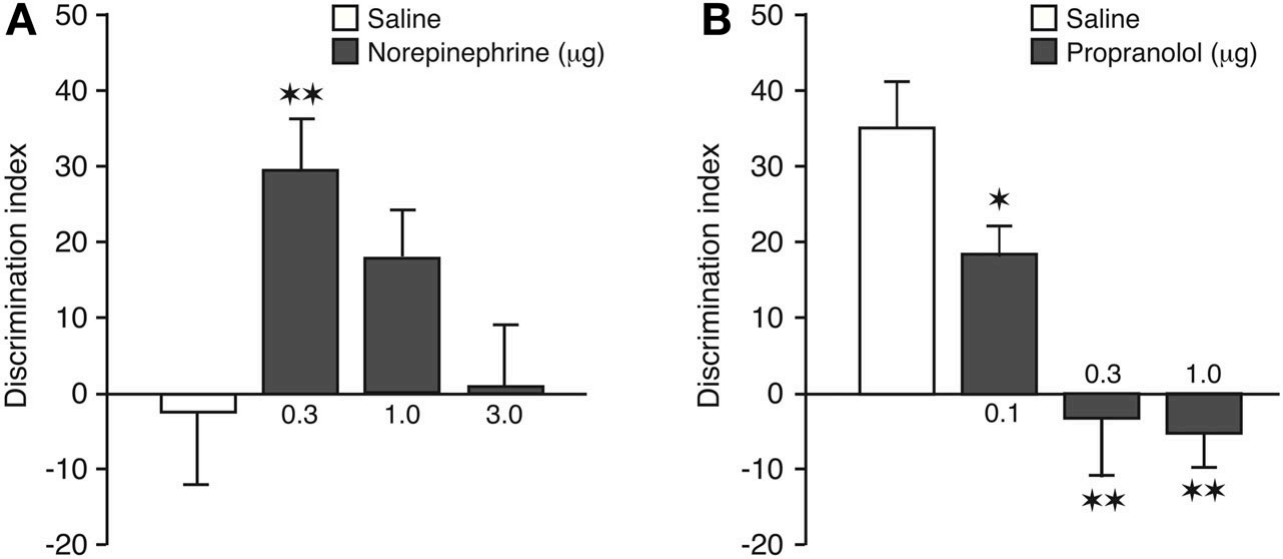
**Noradrenergic activation of the BLA modulates consolidation of object-in-context recognition memory. (A)** Enhancing effects of post-training intra-BLA infusions of norepinephrine on 24-h object-in-context recognition memory. Saline-infused controls displayed no evidence of memory of 3 min of training. The retention performance of the group given 0.3 μg of norepinephrine was significantly better than that of the saline controls. Data are presented as discrimination index (mean ± s.e.m.; see main text). **(B)** Impairing effects of post-training intra-BLA infusions of propranolol on 24-h object-in-context recognition memory. All groups received 10 min of training. Saline-infused controls displayed significant memory and propranolol produced dose-dependent impairment of memory. The performance of all three propranolol groups differed significantly from that of the saline controls. ^*^P < 0.05; ^**^P < 0.01. *N* = 9–15 rats per group.

A one-sample *t*-test revealed that the discrimination index of saline control rats was not significantly different from zero [*t*_(12)_ = −0.22, *P* = 0.83], indicating that they did not express 24-h memory of the object-context configuration. As is shown in Figure 3A, post-training infusions of norepinephrine into the BLA dose-dependently enhanced preference for the familiar object presented in the novel context [One-Way ANOVA: *F*_(3, 48)_ = 3.62; *P* = 0.02]. *Post-hoc* analysis revealed that the discrimination index of rats given the 0.3 μg dose of norepinephrine, but not any of the higher doses, was significantly higher than that of saline-treated rats (*P* < 0.01). Also, one-sample *t*-tests indicated that rats treated with the 0.3 and 1.0 μg doses of nor-epinephrine exhibited a significant exploration preference for the object in the novel context [0.3 μg: *t*_(11)_ = 4.64; *P* = 0.0007; 1.0 μg: *t*_(14)_ = 2.80; *P* = 0.01]. As during the training phase the rats had spent significantly more time exploring the object encountered during the first context exposure, we included object training order, i.e., if the object to be discriminated on the retention test was initially presented in the first or second training context, into the analysis as a separate factor to control for the possibility that retention performance might have been biased by a training order effect. A Two-Way ANOVA for discrimination index revealed a significant drug effect [*F*_(3, 44)_ = 3.74; *P* = 0.02], but no object training order effect [*F*_(1, 44)_ = 1.08; *P*= 0.31] or interaction between these two factors [*F*_(3, 44)_ = 0.64; *P* = 0.59]. Thus, these findings indicate that object training order did not influence retention performance. The saline and nor-epinephrine groups did not differ in total exploration time of the two objects during the retention test [*F*_(3, 48)_ = 2.04; *P* = 0.12; Table 1].

### Effect of propranolol in the basolateral amygdala on memory consolidation of object-in-context training

#### Training session

A Two-Way repeated-measures ANOVA for total object exploration time during the two 10-min context exposures revealed a significant trial effect [*F*_(1, 37)_ = 16.30; *P* = 0.0003], but no difference between later drug treatment groups [*F*_(3, 37)_ = 0.43; *P* = 0.73] or interaction between both factors [*F*_(3, 37)_ = 0.24; *P* = 0.87]. The average time spent exploring the two objects during the first context exposure was 29.2 ± 1.4 s, and 20.7 ± 1.8 s for the second context exposure (paired *t*-test: *P* = 0.0002; *N* = 41), demonstrating that they spent significantly more time exploring the objects during their first context exposure. Rats did not show any difference in their exploration of the two different objects. Table 1 shows the object exploration time during the two context exposures for the different post-training drug treatment groups.

#### Retention test trial

A one-sample *t*-test revealed that the discrimination index of saline control rats was significantly different from zero [*t*_(11)_ = 5.60, *P* = 0.0002], indicating that, in contrast to a 3-min object-context exposure, a 10-min training session results in robust 24-h memory of the object-context configuration. As is shown in Figure 3B, blockade of endogenous noradrenergic activity with post-training infusions of propranolol into the BLA dose-dependently impaired the preference for exploring the familiar object presented in the novel context [One-Way ANOVA: *F*_(3, 37)_ = 10.57; *P* < 0.0001]. *Post-hoc* analysis revealed that the discrimination index of rats given any of the three doses of propranolol was significantly lower than that of saline-treated rats (0.1 μg: *P* < 0.05; 0.3, and 1.0 μg: *P* < 0.01). Also, one-sample *t*-tests indicated that rats treated with the two higher doses of propranolol no longer exhibited an exploration preference for the object presented in the novel context [0.1 μg: *t*_(9)_ = 4.56; *P* = 0.001; 0.3 μg: *t*_(9)_ = −0.37; *P* = 0.72; 1.0 μg: *t*_(8)_ = −1.18; *P* = 0.27]. Similar to the first experiment, rats in the propranolol experiment had spent an unequal time exploring the objects encountered during the two training contexts. Inclusion of object training order into the analyses as a separate factor revealed again that this did not significantly influence the findings. Two-Way ANOVA revealed a significant drug effect [*F*_(3, 33)_ = 10.64; *P* < 0.0001], but no object training order effect [*F*_(1, 33)_ = 0.02; *P* = 0.90] or interaction between these two factors [*F*_(3, 33)_ = 2.19; *P* = 0.11]. The saline and propranolol groups did not differ in total exploration time of the two objects during the retention test [*F*_(3, 37)_ = 0.20; *P* = 0.90; Table 1].

## Discussion

The main finding of the present experiments is that the BLA nor-adrenergic system modulates long-term memory consolidation of object-in-context recognition training, a low-arousing behavioral task designed to assess episodic-like memory in rats.

The present findings indicate that norepinephrine infused into the BLA immediately after 3 min of object-in-context recognition training induced dose-dependent enhancement of 24-h memory of the familiar object presented in a novel context. Hence, while the low degree of arousal produced by 3 min of object-in-context recognition training was insufficient to induce 24-h memory in saline-treated controls, such training conditions were sufficient to enable post-training intra-BLA infusions of nor-epinephrine to induce 24-h memory. As discussed above, most prior studies of the memory-enhancing effects of post-training intra-BLA infusions of norepinephrine have used highly arousing training tasks such as inhibitory avoidance and contextual fear conditioning that use footshock (Introini-Collison et al., 1991; Ferry et al., 1999; LaLumiere et al., 2003; Huff et al., 2005; Berlau and McGaugh, 2006) or aversive water-maze training (Hatfield and McGaugh, 1999). Such findings had suggested that some consequences of high arousal may be essential in enabling nor-adrenergic activation of the BLA to modulate memory consolidation. However, our present findings using the object-in-context recognition paradigm clearly indicate that high levels of training-induced arousal are not required for such modulatory influences. Furthermore, they are consistent with our previous findings of standard object recognition training (Roozendaal et al., 2008) and demonstrate that mildly arousing training conditions coupled with post-training noradrenergic activity within the BLA are sufficient to enable modulation of memory consolidation.

The finding that post-training intra-BLA infusions of propranolol impaired object-in-context recognition memory induced by 10 min of training is consistent with extensive prior evidence that post-training blockade of β-adrenoceptors impairs memory on a variety of highly emotionally arousing training tasks (Liang et al., 1986, 1995; Salinas et al., 1997; Hatfield and McGaugh, 1999). It should be noted that prior studies indicated that post-training inhibition of noradrenergic activity of the BLA had little effect on the consolidation of memory of auditory fear conditioning (Debiec and Ledoux, 2004; Bush et al., 2010). In comparison with the kinds of emotionally arousing training used in previous studies, the training conditions used in the present experiments induced relatively low levels of arousal (Okuda et al., 2004; Roozendaal et al., 2006). Several prior studies using microdialysis and HPLC have reported evidence that norepinephrine release within the amygdala depends on the degree of arousal during or shortly after training. For example, footshock stimulation induces norepinephrine release in the amygdala and the amount varies directly with footshock intensity (Quirarte et al., 1998). Furthermore, 24-h retention of inhibitory avoidance correlates highly with the amount of norepinephrine released following training (McIntyre et al., 2002). We have previously found that object recognition training induces some degree of noradrenergic activation in the BLA, as assessed by an increased immunoreactivity for the phosphorylated form of tyrosine hydroxylase, the rate-limiting enzyme in the synthesis of norepinephrine, within noradrenergic nerve terminals of the BLA. Prior habituation to the training apparatus reduces this noradrenergic activation (Okuda et al., 2004; Roozendaal et al., 2006), indicating that the activation is a consequence of novelty-induced emotional arousal associated with the training procedure. Thus, as in the present experiments rats were not previously habituated to the experimental contexts, it seems likely that the object-in-context recognition training evoked some degree of noradrenergic activation within the BLA. Although there is now extensive evidence that emotional arousal induces noradrenergic modulation of memory consolidation, the present findings indicate that high levels of training-induced arousal are not required in order to observe BLA noradrenergic modulation of memory. Rather, our findings suggest that norepinephrine release in the BLA provides an ongoing modulation of storage of recent experiences that vary in their degree of emotional arousal. Furthermore, our findings provide important additional evidence demonstrating that the amygdala is not exclusively involved in memory processes of fearful or aversive experiences (McGaugh, 2002; Rodrigues et al., 2009).

While it is clear that intense arousal induces highly enduring memories (McGaugh, 2003), in extreme cases potentially contributing to the development of post-traumatic stress disorder (Pitman et al., 2002; McGaugh, 2003), it has also been clearly demonstrated that even very mild arousal enhances episodic (declarative) memory in humans (Phelps, 2004; Labar and Cabeza, 2006). The low-arousing object-in-context task used in the present study might provide the animal analog to the human studies, bridging the gap between the two domains. In agreement with the present findings, propranolol administration to humans has been shown to block the enhanced memory for mildly emotionally arousing material (Cahill et al., 1994). Further, positron emission tomography (PET) and event-related functional magnetic resonance imaging (fMRI) imaging studies have demonstrated that amygdala activity in response to mildly arousing stimuli predicts the subsequent recall of the material (Cahill et al., 1996; Canli et al., 2000). Moreover, pharmacological studies have shown that β-adrenoceptor blockade with propranolol reduces the amygdala response during the encoding of emotionally arousing stimuli (Strange and Dolan, 2004; Van Stegeren et al., 2005; Hurlemann et al., 2010), whereas the selective norepinephrine-reuptake inhibitor reboxetine has an opposite effect (Onur et al., 2009). Other recent studies in humans have shown that amygdala responses during presentation of emotionally arousing stimuli are enhanced in carriers of a deletion variant of *ADRA2B*, the gene encoding the α_2b_-adrenoceptor and resulting in increased noradrenergic tone and enhancement of memory for emotionally arousing stimuli (Rasch et al., 2009). However, as *ADRA2B* deletion carriers did not show enhanced amygdala activity and memory enhancement for emotionally neutral information, some intrinsic arousal appears necessary in order to provide the means by which arousing and non-arousing stimuli become differentiated at this early stage of memory processing. Presumably, this differentiation between emotionally arousing and neutral stimuli is later consolidated into long-term memory.

The majority of human studies investigating the role of the amygdala and noradrenergic system in memory have focused primarily on declarative (episodic) memory tasks. These studies have consistently found enhanced memory of emotionally arousing stimuli, enabling participants' discrimination of otherwise similar stimulus sets and indicating a more distinct separation of memory traces for individual items (Henckens et al., 2009; Smeets et al., 2009; Schwabe and Wolf, 2013; Wiemers et al., 2013). As a result, the subsequently formed memory yields a greater degree of accuracy. The object-in-context paradigm used in our study, adapted from Dix and Aggleton (1999) and Eacott and Norman (2004), also allows the assessment of memory precision, and does so in a naturalistic manner, comparing well with human studies. Some of the parameters from these previous studies were modified, such as the delay between the two training context exposures and the interval between the training and test phase, in order to optimize the task for post-training pharmacological manipulations. Importantly, however, this task incorporates the critical element of contextual discrimination as an episode, similar to that developed by Eacott and Norman (2004) for the assessment of episodic-like memory in rats. While the two presentation episodes were very similar and included a single training trial, the subsequent memory contained sufficient detail to distinguish between them on the retention test. Noradrenergic activation of the BLA after the training trial increased rats' ability to discriminate 24 h later object and context configurations that were different from, yet highly confusable with those presented during training. A number of human studies have found that amygdala activation during learning of declarative (episodic) information influences mnemonic processes that depend on the hippocampus (Cahill et al., 1996, 2001; Hamann et al., 1999; Richardson et al., 2004). Furthermore, β-adrenoceptor blockade with propranolol during memory encoding was shown to diminish the enhancement of hippocampal activity for emotional items at retention testing, when propranolol was no longer active (Strange and Dolan, 2004). Several studies have indicated that object-in-context memory also relies heavily on the hippocampus (Mumby et al., 2002; Balderas et al., 2008), whereas recognition of a novel object itself requires neuronal plasticity in perirhinal and insular cortical regions (Ennaceur and Aggleton, 1997; Bermudez-Rattoni et al., 2005; Albasser et al., 2009; Roozendaal et al., 2010; Banks et al., 2014; Bermudez-Rattoni, 2014). Thus, in further agreement with the memory modulation theory, the present findings clearly suggest that the post-training manipulation of noradrenergic activity within the BLA likely altered memory for this task by changing plasticity in other brain regions and firmly establish that this memory was formed and expressed in the absence of explicit training or motivation, reflecting a naturally formed memory devoid of high levels of arousal.

## Author contributions

Areg Barsegyan, James L. McGaugh, and Benno Roozendaal designed research; Areg Barsegyan performed research; Areg Barsegyan and Benno Roozendaal analyzed data; and Areg Barsegyan, James L. McGaugh, and Benno Roozendaal wrote the paper.

### Conflict of interest statement

The authors declare that the research was conducted in the absence of any commercial or financial relationships that could be construed as a potential conflict of interest.
